# The industrialization of farming may be driving virulence evolution

**DOI:** 10.1111/eva.12442

**Published:** 2016-11-21

**Authors:** Carly Rozins, Troy Day

**Affiliations:** ^1^ Department of Mathematics and StatisticsQueen's UniversityKingstonONCanada

**Keywords:** all‐in‐all‐out, impulsive differential equation, infectious disease, livestock production, mutation, poultry, virulence

## Abstract

Farming practices have changed dramatically over the years. The industrialization of farming has provided parasites with an abundance of hosts and is thought to have influenced parasite evolution. For example, the parasite that causes the highly contagious poultry disease, Marek's disease, has evolved over the past 60 years into a highly virulent pathogen. It is assumed that the industrialization of the industry and vaccination have selected for more virulent strains of the virus. Here, with the use of an impulsive differential equation model, we investigate how modern broiler farm practices could independently lead to virulence evolution. Our model suggests that longer cohort durations and more densely stocked barns both select for less virulent strains of the virus. Our model also suggests that if intensive cleaning between cohorts does not rid the barn of disease, it may drive evolution and cause the disease to become more virulent.

## 1 Introduction

The Marek's disease virus (MDV) is a highly contagious poultry pathogen first recognized over 100 years ago. Up until the 1960s, the disease caused by MDV was characterized as paralytic, occasionally resulting in lymphomas in older birds, and only affecting a low frequency of flocks (Biggs & Payne, 1967). The relatively benign nature of the disease allowed the virus and the chickens to coexist without the necessity of human intervention.

In the early 1960s, there was a widespread move from small‐scale farming to intensive large‐scale farming. For the first time, large flocks of chickens were being reared together in crowded farms. It is believed that these changes in husbandry practices caused an evolutionary shift towards greater virulence in Marek's disease (MD) (Nair, 2005). The disease continued to cause paralysis, but was now accompanied by an unusually large presence of lymphomas in very young birds. This resulted in widespread outbreaks and very high mortality rates around the world (Benton & Cover, 1957; Witter, 1997). There was an urgent need for human intervention to combat economic losses, and the solution came in the form of vaccination (Churchill, Payne, & Chubb, 1969; Okazaki, Purchase, & Burmester, 1970). Vaccination campaigns were so successful that deaths due to MD became almost nonexistent (Witter, 2001).

In the 1970s, the vaccine in circulation started to loose efficacy and infections with more virulent strains of the MDV were observed. As losses due to MD started to dramatically increase again, the poultry industry responded with a new bivalent vaccine that offered better protection from the strains in circulation. The bivalent vaccine was successful, but not for long. By the mid 1990s, there was another shift in the disease towards higher virulence and an ability to bypass the bivalent vaccine‐induced immune response (Nair, 2005; Witter, 1998). Again the industry combatted the escalating losses due to disease with a new more potent vaccine. The new vaccine, like the previous ones, offered immediate relief, but there is concern that this protection will again be temporary (Nair, 2005).

There has never been, nor is there now, a treatment for MD, and case fatality risk can be as high as 100% in unvaccinated birds (Nair, 2005; Witter, 1998). The only defence against MD is vaccines, which are administered to chicks on the day of hatching or in ovo. In ovo vaccination automates the process and allows the chickens to develop immunity as early as possible (Sarma, Greer, Gildersleeve, Murray, & Miles, 1995). The MD vaccines have all been “imperfect vaccines”, which are unable to induce a sterile immunity in the vaccinated host. The vaccine protects the host from developing signs associated with the disease, but does not prevent infection and will allow the virus to replicate within the host (Purchase & Okazaki, 1971). Therefore, vaccinated chickens can become infected with MDV, and once infected, they can transmit it. In addition, once MDV makes its way into a barn, the disease may go undetected because the vaccine masks the disease. The MDV is transmitted indirectly through the inhalation of viral particles, which are shed by infected birds (Witter, Moulthrop, Burgoyne, & Connell, 1970). MDV has been found in depopulated barns (Jurajda & Klimes, 1970), and it has been shown to cause infection weeks after being shed (Carrozza, Fredrickson, Prince, & Luginbuhl, 1973; Jurajda & Klimes, 1970; Witter, Burgoyne, & Burmester, 1968). Therefore, a chicken can become infected by viral particles shed by a bird from a previous flock.

It has been suggested that the industrialization of the industry in the 1960s led to an increase in virulence (Nair, 2005). The emergence of more virulent strains since the 1970s has largely been attributed to the widespread use of vaccinations (Davison & Nair, 2005), and this has been supported by empirical and theoretical work (Atkins et al., 2012; Read et al., 2015). Atkins et al. (2012) constructed a model of MDV fitness for a range of vaccination treatments and cohort durations. They used an individual‐based approach in deriving an expression for pathogen fitness and with it found that the reduced lifespan of poultry on industrial farms (reduced cohort duration) and the introduction of vaccination can lead to an increase in virulence. However, these results stem from a single‐cohort model and therefore have ignored all intercohort dynamics, such as the cleaning and restocking of the barn. By ignoring intercohort dynamics, the survival of free‐living viral particles between subsequent cohorts of chickens is completely overlooked.

In this article, we do not directly investigate vaccination, but instead explore how the current state of the industry can select for more or less virulent strains of the virus. In particular, we look at the duration of time a chicken spends in a barn, the number of chickens reared together in the barn and the cleaning and restocking regimen of the barn. We develop a mathematical model, which tracks the spread of MD on a single industrial broiler farm. We capture both the within‐cohort dynamics (rearing of the chickens) and the intercohort dynamics (cleaning and restocking of the barn) with the use of an impulsive differential equation model. We model the current state of MD in which vaccination provides chicks with an effective immune response against the circulating strains of the virus, which we refer to as wild‐type strains. This model differs from other mathematical models of poultry diseases (Atkins, Read, Walkden‐Brown, Savill, & Woolhouse, 2013; Atkins et al., 2012; Klinkenberg & Heesterbeek, 2005), in that it models long‐term persistence of disease on a single farm.

## 2 Model

The spread of MDV between chickens on a farm is modelled with a compartmental model. The chicken population is split into three subpopulations: susceptible, infected with the wild‐type strain and infected with the mutant strain. The number of susceptible chickens is denoted *S*, the number of wild‐type infected chickens is denoted *I*_1_, and the number of chickens infected with a mutant virus is denoted *I*_2_. We assume that there is no superinfection or co‐infection. Susceptible individuals can become infected following contact with viral particles, which are shed by infected birds.

The density of wild‐type viral particles is denoted *F*_1_, and the density of mutant viral particles is denoted *F*_2_. The rate of infection of susceptible chickens is governed by the transmission rates σ_1_ and σ_2_ and is proportional to the current susceptible population size as well as either the density of wild‐type viral particles in the barn or the density of mutant viral particles in the barn at that time. Chickens infected with the wild‐type strain shed viral particles at rate κ_1_, and chickens infected with the mutant strain shed viral particles at a rate κ_2_. Once a chicken perishes, which occurs at a rate *v*_1_ for chickens infected with the wild‐type strain and *v*_2_ for chickens infected with the mutant strain, ω_1_ or ω_2_ viral particles are released into the barn. We assume that there is no natural death and that *v*_1_ is the wild‐type strain disease‐induced death rate and *v*_2_ is the mutant strain disease‐induced death rate.

Our model assumes that every chicken has been vaccinated and that the vaccine is highly effective at preventing the wild‐type strain from causing disease and killing its host (*v*_1_ is assumed to be small). The viral particles leave the barn either through natural decay or through ventilation systems at rate δ_1_ for wild‐type viral particles, and δ_2_ for mutant viral particles. The system of differential equations that model the spread of MDV between chickens on a broiler farm is referred to as the *within‐cohort dynamics* and is given by equations ((1a), (1b), (1c), (1d), (1e)).

The *intercohort dynamics*, equations ((1f), (1g), (1h), (1i), (1j)), model the emptying and restocking of the barn. On day *nT*, where *n* = 1, 2, …, the broiler farm is emptied (the chickens go to slaughter), it is cleaned and then restocked with *N* new susceptible chickens. Because the time it takes to empty, clean and restock the farm is small relative to the cohort duration, we treat this intercohort period as occurring during an instant in time. Thus, we use the notation *nT* ^−^ and *nT* ^+^ to denote the times immediately before and after the change of cohort, respectively. The population numbers at the end of a cohort, directly before the moment of impulse, are determined by the differential equations ((1a), (1b), (1c), (1d), (1e)). The population numbers directly after an impulse are determined by equations ((1f), (1g), (1h), (1i), (1j)) and act as initial conditions for the next cohort. The proportion of wild‐type viral particles and mutant viral particles that remain on the farm after it has been cleaned (after the impulsive condition) are denoted 0 ≤ γ_1_ ≤ 1 and 0 ≤ γ_2_ ≤ 1, respectively.

### 2.1 Impulsive model

By combining both the continuous dynamics, equations ((1a), (1b), (1c), (1d), (1e)), and the discrete mapping, equations ((1f), (1g), (1h), (1i), (1j)), we arrive at an impulsive set of differential equations, capable of tracking the spread and persistence of two strains of the MDV in a barn over an indefinite number of cohorts. For *T* days, the model is described by equations ((1a), (1b), (1c), (1d), (1e)), after which it is governed by equations ((1f), (1g), (1h), (1i), (1j)) for an instant change in state, which models the emptying, cleaning and restocking.

(1a) $$\frac{dS(t)}{dt}=-\sigma_{1}S\left(t\right)F_{1}\left(t\right)-\sigma_{2}S\left(t\right)F_{2}\left(t\right),$$

(1b) $$\frac{dI_{1}\left(t\right)}{dt}=\sigma_{1}S\left(t\right)F_{1}\left(t\right)-v_{1}I_{1}\left(t\right),$$

(1c) $$\frac{dI_{2}\left(t\right)}{dt}=\sigma_{2}S\left(t\right)F_{2}\left(t\right)-v_{2}I_{2}\left(t\right)\;\;t\;\neq \;nT,\;\text{for}\;n=1,\;2,\ldots ,$$

(1d) $$\frac{dF_{1}\left(t\right)}{dt}=\kappa_{1}I_{1}\left(t\right)+v_{1}\omega_{1}I_{1}\left(t\right)-\delta_{1}F_{1}\left(t\right),$$

(1e) $$\frac{dF_{2}\left(t\right)}{dt}=\kappa_{2}I_{2}\left(t\right)+v_{2}\omega_{2}I_{2}\left(t\right)-\delta_{2}F_{2}\left(t\right),$$

(1f) $$S(nT^{+})=N,$$

(1g) $$I_{1}\left(nT^{+}\right)=0,$$

(1h) $$I_{2}\left(nT^{+}\right)=0\;t=nT,\;\text{for}\;n=1,\;2,\ldots ,$$

(1i) $$F_{1}\left(nT^{+}\right)=\gamma_{1}F_{1}\left(nT^{-}\right),$$

(1j) $$F_{2}\left(nT^{+}\right)=\gamma_{2}F_{2}\left(nT^{-}\right).$$

### 2.2 Reduced impulsive model

In most cases, the rate of change of the viral particle population is fast relative to the dynamics of the susceptible and infected chickens. Consequently, we separate the dynamics into “fast” and “slow” components. In particular, we suppose that the viral particle densities (both mutant and wild‐type) in the barn reach a quasi‐equilibrium quickly during the within‐cohort dynamics, and this thereby allows us to reduce equations ((1a), (1b), (1c), (1d), (1e)), to three equations. If we define the composite parameters β_1_ = σ_1_(κ_1_ + *v*_1_ω)/δ_1_ and β_2_ = σ_2_(κ_2_ + *v*_2_ω)/δ_2_, then equations ((1a), (1b), (1c), (1d), (1e)) reduce to:

(2) $$\begin{array}{cc} & \frac{dS(t)}{dt}=-\beta_{1}S\left(t\right)I_{1}\left(t\right)-\beta_{2}S\left(t\right)I_{2}\left(t\right)\\ & \frac{dI_{1}\left(t\right)}{dt}=\beta_{1}S\left(t\right)I_{1}\left(t\right)-v_{1}I_{1}\left(t\right)\\ & \frac{dI_{2}\left(t\right)}{dt}=\beta_{2}S\left(t\right)I_{2}\left(t\right)-v_{2}I_{2}\left(t\right) \end{array}$$

where we can view β_1_ and β_2_ as transmission rates.

To proceed further, we also need to reduce the intercohort jump in state, given by equation ((1f), (1g), (1h), (1i), (1j)), to three equations in *S*,* I*_1_ and *I*_2_. In other words, we need to translate the number of viral particles remaining at the end of a cohort, *F*_1_(*nT* ^−^) and *F*_2_(*nT* ^−^), into a number of susceptible and infected chickens at the start of the next cohort, *S*(*nT* ^+^), *I*_1_(*nT* ^+^) and *I*_2_(*nT* ^+^). To do so, we will explicitly model a process of viral particle decay and infection during the intercohort period. However, we will still suppose that this intercohort period is very short compared to the duration of a cohort.

During the intercohort period, the viral particles will decay exponentially at per capita rates δ_1_ and δ_2_ for wild‐type and mutant particles, respectively. Therefore, using $\phi_{1}\left(\tau \right)\;\text{and}\;\phi_{2}\left(\tau \right)$ to denote the number of wild‐type and mutant particles remaining at time τ during the intercohort period, we have

$$\phi_{1}\left(\tau \right)=\gamma_{1}F_{1}\left(nT^{-}\right)e^{-\delta_{1}\tau }\;\;\text{and}\;\;\phi_{2}\left(\tau \right)=\gamma_{2}F_{2}\left(nT^{-}\right)e^{-\delta_{2}\tau },$$

where we have assumed that time τ = 0 corresponds to immediately after cleaning. Based on our quasi‐equilibrium assumption, the density of viral particles in the barn at the end of a cohort is given by $F_{1}\left(nT^{-}\right)=I_{1}\left(nT^{-}\right)\left(\kappa_{1}+v_{1}\omega_{1}\right)/\delta_{1}=\frac{\beta_{1}}{\sigma_{1}}I_{1}\left(nT^{-}\right)$ and $F_{2}\left(nT^{-}\right)=I_{2}\left(nT^{-}\right)\left(\kappa_{2}+v_{2}\omega_{2}\right)/\delta_{2}=\frac{\beta_{2}}{\sigma_{2}}I_{2}\left(nT^{-}\right)$. Therefore, $\phi_{1}\left(\tau \right)\;\text{and}\;\phi_{2}\left(\tau \right)$ can be written as

$$\phi_{1}\left(\tau \right)=\gamma_{1}\frac{\beta_{1}}{\sigma_{1}}I_{1}\left(nT^{-}\right)e^{-\delta_{1}\tau }\;\;\text{and}\;\;\phi_{2}\left(\tau \right)=\gamma_{2}\frac{\beta_{2}}{\sigma_{2}}I_{2}\left(nT^{-}\right)e^{-\delta_{2}\tau }.$$

Now we suppose that a new cohort of completely susceptible chickens is moved into the barn at time τ = 0, and some chickens become infected during this intercohort period, as the population of viral particles rapidly decay. Specifically, we suppose that $dS/d\tau =-\sigma_{1}\phi_{1}\left(\tau \right)S\left(\tau \right)-\sigma_{2}\phi_{2}\left(\tau \right)S\left(\tau \right)$ during this period. Thus, solving this equation, we see that at time τ during the intercohort period, the number of susceptible chickens is

$$S\left(\tau \right)=Ne^{-\frac{\gamma_{1}\beta_{1}}{\delta_{1}}I_{1}\left(nT^{-}\right)\;-\frac{\gamma_{2}\beta_{2}}{\delta_{2}}I_{2}\left(nT^{-}\right)}e^{\frac{\gamma_{1}\beta_{1}}{\delta_{1}}I_{1}\left(nT^{-}\right)e^{-\delta_{1}\tau }\;+\;\frac{\gamma_{2}\beta_{2}}{\delta_{2}}I_{2}\left(nT^{-}\right)e^{-\delta_{2}\tau }}.$$

As the decay of viral particles is fast (i.e., δ_1_ and δ_2_ are large), the number of susceptible chickens remaining at the end of the intercohort phase can be approximated as

$$Ne^{-\frac{\gamma_{1}\beta_{1}}{\delta_{1}}I_{1}\left(nT^{-}\right)-\frac{\gamma_{2}\beta_{2}}{\delta_{2}}I_{2}\left(nT^{-}\right)}.$$

Furthermore, if very few chickens become infected during this intercohort phase (i.e., $\frac{-\gamma_{1}\beta_{1}}{\delta_{1}}I_{1}\left(nT^{-}\right)+\frac{-\gamma_{2}\beta_{2}}{\delta_{2}}I_{2}\left(nT^{-}\right)$ is relatively small), then we can further approximate this as

$$N\left(1-\frac{\gamma_{1}\beta_{1}}{\delta_{1}}I_{1}\left(nT^{-}\right)-\frac{\gamma_{2}\beta_{2}}{\delta_{2}}I_{2}\left(nT^{-}\right)\right).$$

We therefore take this to be the number of susceptible chickens at the beginning if the next cohort, *S*(*nT* ^+^). Likewise, the total number of infected chickens at the beginning of the next cohort is approximately:

$$I_{1}\left(nT^{+}\right)=N\frac{\gamma_{1}\beta_{1}}{\delta_{1}}I_{1}\left(nT^{-}\right)\;\;\text{and}\;\;I_{2}\left(nT^{+}\right)=N\frac{\gamma_{2}\beta_{2}}{\delta_{2}}I_{2}\left(nT^{-}\right).$$

Again, treating the intercohort period as being very short relative to the cohort duration, we then arrive at a three‐dimensional impulsive differential equation model, given by:

(3) $$\begin{array}{cc} \frac{dS}{dt} & =-\beta_{1}S\left(t\right)I_{1}\left(t\right)-\beta_{2}S\left(t\right)I_{2}\left(t\right)\\ \frac{dI_{1}}{dt} & =\beta_{1}S\left(t\right)I_{1}\left(t\right)-v_{1}I_{1}\left(t\right)\;t\;\neq \;nT,\;\text{for}\;n=1,\;2,\ldots \\ \frac{dI_{2}}{dt} & =\beta_{2}S\left(t\right)I_{2}\left(t\right)-v_{2}I_{2}\left(t\right)\\ S(nT^{+}) & =N-a_{1}I_{1}\left(nT^{-}\right)-a_{2}I_{2}\left(nT^{-}\right)\\ I_{1}\left(nT^{+}\right) & =a_{1}I_{1}\left(nT^{-}\right)\;t=nT,\;\text{for}\;n=1,\;2,\ldots \\ I_{2}\left(nT^{+}\right) & =a_{2}I_{2}\left(nT^{-}\right) \end{array}$$

where *a*_1_ = *N*γ_1_β_1_/δ_1_ << 1 and *a*_2_ = *N*γ_2_β_2_/δ_2_ << 1.

## 3 Results

We begin our analysis of (3) by first demonstrating the existence of a “mutant‐free” solution, in which mutant infections are entirely absent from the population (i.e., *I*_2_(*t*) = 0 for all *t*), and the wild‐type strain is endemic. We define as follows:

$$\Lambda =a_{1}e^{N\beta_{1}T}-\frac{v_{1}a_{1}\left(e^{N\beta_{1}T}-1\right)}{\beta_{1}N}.$$

**Figure 1:**
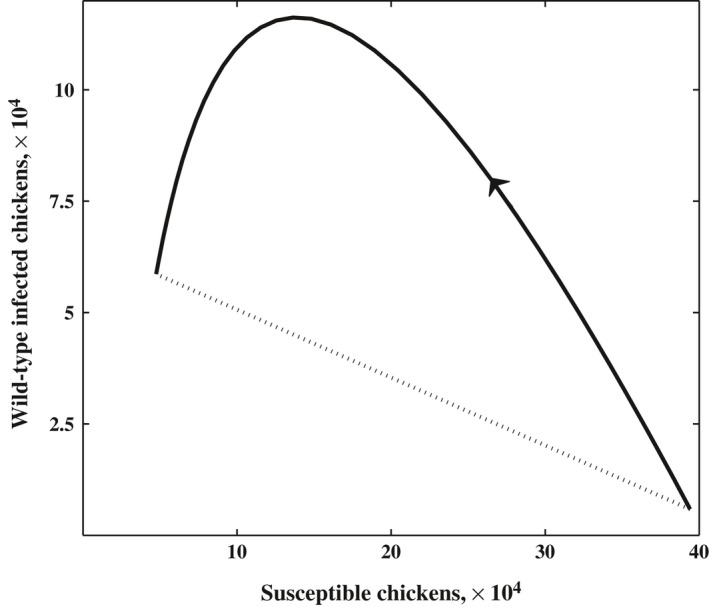
Phase portrait of model (3) with *I*_2_(*t*) = 0 for all *t* and Λ > 1. The stable periodic orbit is jump continuous, with the jump highlighted by the dashed line. The arrow shows the direction of the orbit. We have set the parameter values *N* = 40,000, *T* = 42, *a*_1_ = 0.1, *v*_1_ = 0.1 and β_1_ = 7.2 × 10^−6^. The parameter values *N* and *T* are taken from the literature (Sheppard, 2004), while all other parameter values are fixed at values that satisfy Λ > 1

If 0 < *v*_1_ << 1 and Λ > 1, then the wild‐type strain, in the absence of the mutant strain, is endemic in the population and the population densities described by (3) converge to stable periodic orbits (see Figure 1 and (Rozins & Day, 2016) for proof). The functions describing the periodic orbits are denoted S*(*t*) and $I_{1}^{*}\left(t\right)$ for the susceptible and wild‐type‐infected populations, respectively, and we will denote the periodic solution in the absence of mutants as (S*(*t*), I*(*t*), 0). Once at the stable periodic solution, the number of susceptible chickens and the number of infected chickens at the start of every cohort will be constant (i.e., S*(*nT* ^+^) = S*((*n* + 1)*T* ^+^) and $I_{1}^{*}\left(nT^{+}\right)\;=\;I_{1}^{*}\left(\left(n+1\right)T^{+}\right)$ for all *n*). Also note that the exact equations describing the periodic solutions S*(*t*) and $I_{1}^{*}\left(t\right)$ will depend on the initial conditions *S*(0) and *I*_1_(0).

### 3.1 Mutant invasion

Suppose that infections with the mutant strain of the virus are completely absent from the barn, and that infections with the wild‐type strain are endemic and have reached a stable periodic orbit (i.e., Λ > 1, *v*_1_ << 1, *S*(*t*) = S*(*t*), $I_{1}\left(t\right)\;=\;I_{1}^{*}\left(t\right)$ and *I*_2_(*t*) = 0). Now suppose that at time *t* = *mT* ^+^, the start of the *mT*th cohort, (*m* = *n* − 1), a mutant strain of the virus emerges in the barn, and the density of chickens infected with the mutant strain is denoted *I*_2_(*mT* ^+^). We assume *I*_2_(*mT* ^+^) is very small relative to the flock size. This mutant strain will fail to invade if the number of chickens infected with the mutant strain decreases from the *mT*th cohort to the (*m* + 1)*T*th cohort:

(4) $$I_{2}\left(mT^{+}\right)>I_{2}\left(\left(m+1\right)T^{+}\right).$$

The introduction of a small number of chickens infected with the mutant strain acts as a perturbation to the periodic orbit (S*(*t*), I*(*t*), 0). An evolutionary stable strategy (ESS) is a strategy which, if adopted by a population in a given environment, cannot be invaded by any alternative strategy that is initially rare. Therefore, the wild‐type strain is an ESS if (S*(*t*), I*(*t*), 0) is stable in this perturbed system. We can use inequality (4) to characterize the ESS.

Consider the third differential equation in (3). If we integrate over the *mT*th cohort duration, we can determine the number of chickens infected with the mutant strain of the virus at the end of the cohort:

$$I_{2}\left(\left(m+1\right)T^{-}\right)=I_{2}\left(mT^{+}\right)e^{\beta_{2}\int_{mT^{+}}^{\left(m+1\right)T^{-}}S\left(t\right)\;dt\;-\;v_{2}T}.$$

Using the sixth equation in (3), the above can be then transformed into the number of chickens infected with the mutant strain of the virus, directly after the moment of impulse,

(5) $$I_{2}\left(\left(m+1\right)T^{+}\right)=a_{2}I_{2}\left(mT^{+}\right)e^{\beta_{2}\int_{mT^{+}}^{\left(m+1\right)T^{-}}S\left(t\right)\;dt\;-\;v_{2}T}.$$

For the mutant virus to fail to invade, we therefore require:

(6) $$\int_{mT^{+}}^{\left(m+1\right)T^{-}}S\left(t\right)\;dt<\frac{Tv_{2}-\text{ln}\left(a_{2}\right)}{\beta_{2}}.$$

Now consider the second differential equation in (3), which describes the rate of change of chickens infected with the wild‐type strain during a cohort. If we divide both sides by *I*_1_(*t*) and integrate, we arrive at:

(7) $$\text{ln}(\frac{I_{1}\left(\left(m+1\right)T^{-}\right)}{I_{1}{\left(mT\right)}^{+}})=\beta_{1}\int_{mT^{+}}^{\left(m+1\right)T^{-}}S\left(t\right)\;dt-v_{1}T.$$

It is assumed that the introduction of chickens infected with the mutant strain of the virus will leave the periodic obit, $I_{1}^{*}\left(t\right)$, unchanged over the first cohort duration, and thus, *I*_1_(*mT* ^+^) = *I*_1_((*m* + 1)*T* ^+^), the number of chickens infected with the wild‐type strain, at the start of the second cohort, is exactly the number of chickens infected with the wild‐type strain at the start of the initial cohort. Therefore, *I*_1_((*m* + 1)*T* ^+^) = *I*_1_(*mT* ^+^) = *a*_1_ *I*_1_((*m* + 1)*T* ^−^) and $\frac{I_{1}\left(\left(m+1\right)T^{-}\right)}{I_{1}\left(mT^{+}\right)}=\frac{1}{a_{1}}$. Thus, (7) can be rewritten as,

$$\int_{mT^{+}}^{\left(m+1\right)T^{-}}S\left(t\right)\;dt=\frac{v_{1}T-\text{ln}\left(a_{1}\right)}{\beta_{1}},$$

**Figure 2:**
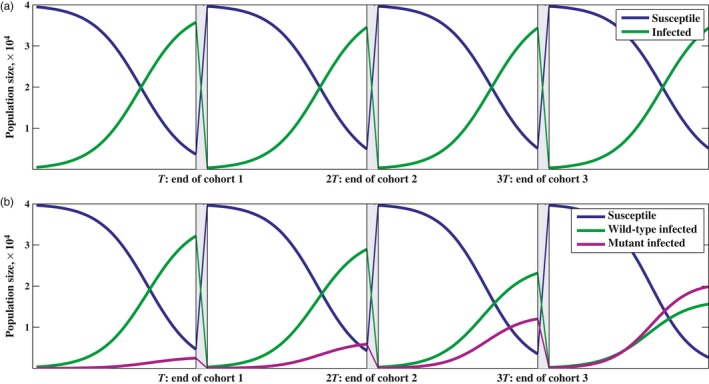
Numerical results for model (3). (a) The mutant‐free system converging to the stable periodic orbit. Note that near the stable periodic orbit, each cohort will start with approximately the same number of susceptible chickens and the same number of infected chickens as the previous cohort. We have set β_1_ = 4.0 × 10^−6^, *v*_1_ = 0.001, *T* = 42, *N* = 40,000, *a*_1_ = 0.01 (b) The rare mutant strain invading and replacing the resident strain. β_1_ = 4.0 × 10^−6^, β_2_ = 5.0*e* − 06, *v*_1_ = 0.001, *v*_2_ = 0.005, *T* = 42, *N* = 40,000, *a*_1_ = 0.01, *a*_2_ = 0.01. Note that the interval width at the moments of impulse *T*, 2*T* and 3*T* are exaggerated, and not to scale, in an effort to emphasize the intercohort dynamics

If we substitute the above into (6), we get:

(8) $$\frac{\beta_{1}}{v_{1}-\frac{\text{ln}(a_{1})}{T}}>\frac{\beta_{2}}{v_{2}-\frac{\text{ln}(a_{2})}{T}}$$

Therefore, the wild‐type strain can withstand invasion by any other strain provided that inequality (8) is satisfied. This means that the ESS is the strain that maximizes:

(9) $$\frac{\beta }{v-\frac{\text{ln}(a)}{T}}.$$

### 3.2 The evolutionary stable virulence level

Expression (9) is a measure of pathogen fitness. The numerator in (9) is a measure of pathogen transmission and can be viewed as the benefit of an increase in virulence, while the denominator is a measure of host death rate and can be viewed as the cost of virulence. The disease‐induced death rate is *v*, while −ln (*a*)/*T* can be thought of as an effective death rate due to cleaning and loss during the intercohort period. Over the intercohort period, a proportion, *a*, of the infected chickens survive while the rest die. This can be viewed as an instant of mortality for the pathogen, but suppose instead that this instant of mortality was instead spread out over the cohort period as a constant mortality rate, μ. The proportion of pathogens alive at the end of a cohort is *e* ^−μ*T*^; therefore, μ = −ln (*a*)/*T*.

It is assumed that the amount of pathogen within the chicken is related to the level of virulence of the disease. Higher levels of virulence result from a higher density of pathogen within the infected chicken, and as a result, this will affect the shedding rate and hence the transmission rate. Therefore, it is assumed that the shedding rate, κ(*v*), is a function of virulence, *v*. This assumption is supported by Atkins et al. (2011), who found that the long‐term shedding rate is higher for strains of higher virulence. Recall that:

$$\beta =\frac{\sigma (\kappa +v\omega )}{\delta }\;\;\text{and}\;\;a=\frac{N\gamma }{\delta }\beta ,$$

As the transmission rate, β, is a function of κ, and κ is a function of virulence level, we can write the transmission rate as a function of virulence, β(*v*). Also if we let *k* = *N*γ/δ, we can write *a* = *k*β(*v*). Therefore, (9) can be rewritten as:

(10) $$\frac{\beta (v)}{v-\frac{\text{ln}(k\beta (v))}{T}}.$$

As the function κ(*v*) is unknown, so too is the function β(*v*), but we assume that it is an increasing function of *v* and concave down. This is the standard transmission–virulence trade‐off assumption (Alizon, Hurford, Mideo, & Van Baalen, 2009). In other words, increases in transmission can only come at a “cost” of an increase in virulence.

If we take the first derivative of (10), we arrive at:

(11) $$\frac{d\beta (v^{*})}{dv}=\frac{\beta (v^{*})}{v^{*}+\mu \left(v^{*}\right)+\frac{1}{T}},$$

where $\mu \left(v^{*}\right)=-\frac{1}{T}\text{ln}\left(k\beta \left(v^{*}\right)\right)$ and v* is the ES virulence level, which maximizes (10). An equation very similar to (11) can be derived from the classical epidemiological model used to study virulence evolution. A classical model that includes terms for transmission, β, pathogen‐induced death, *v*, and background host mortality, μ, has a reproductive ratio of:

$$R_{0}=\frac{\beta (v)}{v+\mu }.$$

The reproductive ratio is a measure of pathogen fitness. By maximizing *R*_0_ (i.e., taking the first derivative and setting it equal to zero), we arrive at,

(12) $$\frac{d\beta (v^{*})}{dv}=\frac{\beta (v^{*})}{v^{*}+\mu },$$

an equation very similar to (11). Both (11) and (12), along with the transmission–virulence trade‐off curve, can provide insights into how virulence evolves with changes to background host mortality, μ, or in the case of (11), cohort duration.

In Figure (2), we illustrate how virulence evolution can be inferred, geometrically, from the transmission–virulence trade‐off curve. The left hand side of (12) is the slope of the transmission–virulence trade‐off curve at the ES virulence level. Therefore, for a fixed background host mortality, μ, the ES virulence level can be found by drawing a line from μ, tangent to the curve β(*v*). This line will have slope β(v*)/(v* + μ). The point of tangency corresponds to the ES virulence level, v*, for that fixed background host mortality, μ. As can been seen in Figure (2), as the value of μ decreases, the slope of the line tangent to the curve, β(*v*), increases, and thus v* decreases.

**Figure 3:**
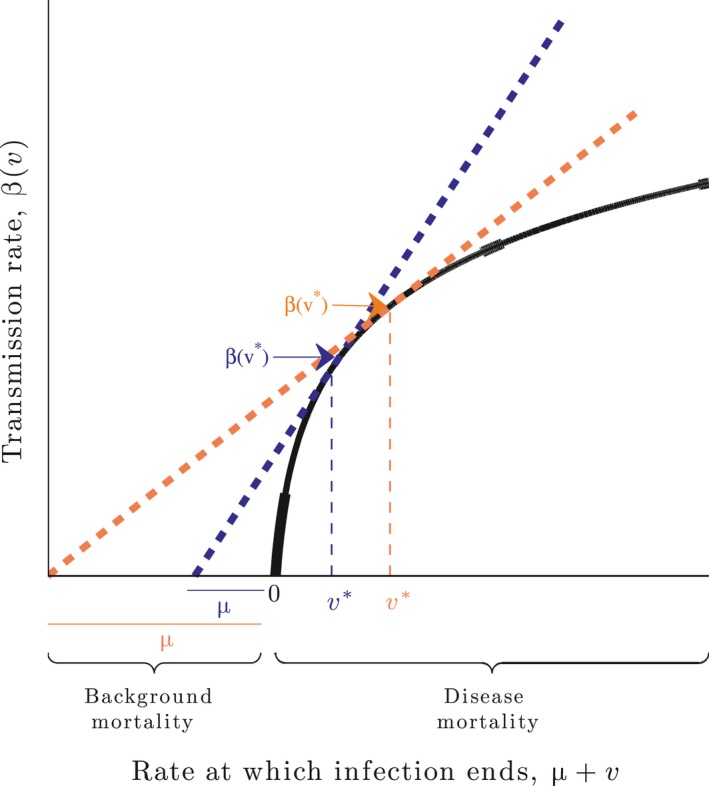
The transmission–virulence trade‐off curve (in black) is the boundary of all possible combinations of transmission and virulence. Given a fixed background death rate, μ, evolution will favour a transmission and virulence combination that maximizes parasite fitness. We can geometrically predict virulence evolution from the trade‐off curve. The derivative β′(v*) = β(v*)/(v* + μ), (12), is the slope of the curve, β(*v*), at the ES virulence level, v*. It is the ratio of transmission (on the *y*‐axis) over the duration of infection (on the *x*‐axis). Therefore, for fixed μ, the ES virulence level can be found by drawing a line from μ, tangent to the curve. The point along the line, tangent to the curve, is v*. Consider the orange line, which is the slope of β(*v*) at the second (larger) v* value. This slope corresponds to a greater μ value. As μ decreases, the slope of the line (now the blue line) increases and the point at which the line is tangent to the curve β(*v*) is farther to the left, and this point corresponds to a smaller ES virulence level. In other words, a decrease in background host mortality decreases virulence

#### 3.2.1 Cohort duration

**Figure 4:**
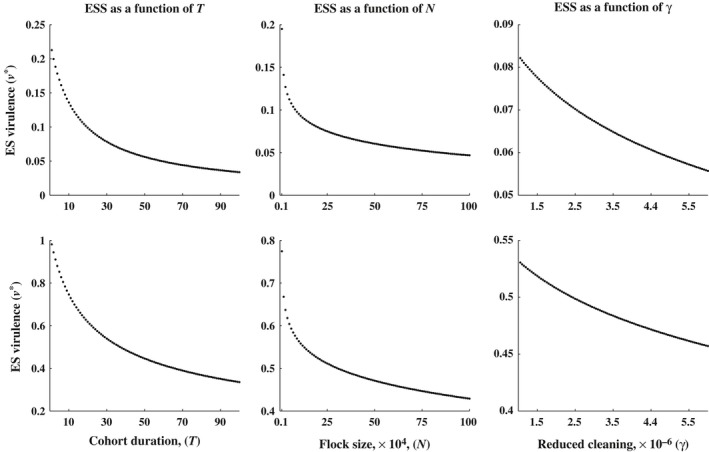
Evolutionary stable virulence level, v*, for varying levels of *T*,* N*, γ and functions of κ(*v*). Each row of plots assumes the same function for κ. The plots are produced by maximizing (9). Top row: $\kappa \left(v\right)=\sqrt{40v}$ and in the bottom row: κ(*v*) = ln (*v* + 1). In each column of plots, we investigate 100 different values of that parameter value (*T*,* N* or γ). For each of the 100 values per plot, we hold all parameters fixed while we solve for v*. Increases in *T*,* N* and γ all lead to lower ES virulence levels. Parameters are fixed at ω = 0.03, σ = 0.02, δ = .5, γ = 0.15, *N* = 40,000 and *T* = 42 unless otherwise stated

**Figure 5:**
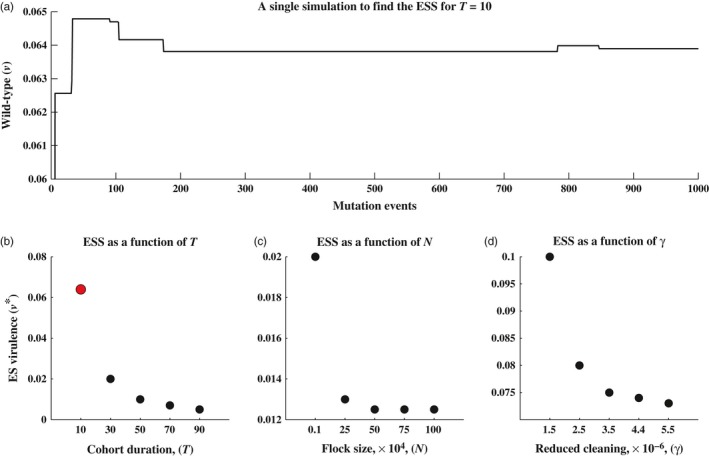
Simulations for determining the evolutionary stable virulence level, v*, for varying levels of *T*,* N* and γ. In plot (a), we find the evolutionarily stable virulence level for *T* = 10. Using model (1), we sequentially introduce 1,000 unique randomly chosen mutants into the barn. The solid line is the current wild‐type strain of MD in the barn. The line jumps up or down when a mutant strain successfully invades and becomes the new wild‐type strain. After investigating the 1,000 random mutants, we have a good approximation for the ES virulence level, and we have produced the single red dot in plot (b). Each dot in figures (b–d) is found by performing a similar simulation to the one in plot (a), but for varying values of *T*,* N* and γ. All other parameters values are held at ω = 0.03, σ = 0.02, $\kappa_{i}=\sqrt{40v_{i}}$, δ = 0.5, γ = 0.15, *N* = 40,000 and *T* = 42, unless otherwise stated

Increasing the cohort duration, *T*, decreases the ES virulence level, v* (see Figures 3, 4, 5, and the Appendix S1). This relationship is illustrated in Figure (2), where we have a typical trade‐off curve for virulence evolution. For a fixed cohort duration, evolution will favour a transmission and virulence combination that maximizes parasite fitness (10). As the cohort duration increases, the effective death rate, μ(v*), decreases, and as a result, the slope of the line tangent to the curve, β(*v*), will increase, corresponding to a smaller v* value (see Figure 3). Biologically, this relationship is due to the increase in parasite life expectancy, given an increase in cohort duration.

As the cohort duration increases, the lifespan of the chicken increases, and thus, the life span of and MDV within the chicken is also extended. With an increase in life span, the parasite's ESS will transition to become less virulent, a strategy better suited for a longer life span. Therefore, increasing the cohort duration, *T*, is similar to decreasing background host mortality, which has traditionally been shown to select for lower virulence (Cressler, McLeod, Rozins, Van Den Hoogen, & Day, 2016).

#### 3.2.2 Flock size and cleaning effort

The evolutionary stable virulence level, v*, decreases with an increase in flock density (an increase in *N*), and/or a decrease in cleaning (increase in γ; see Figures 3, 4, 5 and the Appendix S1). This relationship is illustrated in Figure (2); as *N* or γ increase, the effective death rate, μ(v*), decreases. This results in the line tangent to the curve, β(*v*), becoming more steep, which implies the ES virulence level, v*, decreases. Biologically, the increase in virulence is due to an increase in parasite life span that results from an increase in *N* or γ.

Increasing the density of chickens, (i.e., increasing *N*), or decreasing the cleaning effort between cohorts, (i.e., increasing γ), increases parasite survival throughout the intercohort period and thus increases the life span of the pathogen (i.e., increasing *N* and/or γ, increases 1/(*v* − ln (*k*β(*v*))/*T*)). In traditional models of virulence evolution, this is a similar effect to a decrease in background host mortality (Cressler et al., 2016), and hence why we have referred to μ(*v*) = ln (*k*β(*v*))/*T* as an effective death rate. As background host mortality increases, the lifespan of the pathogen decreases, and thus, the cost of being very virulence decreases.

#### 3.2.3 Extensions and robustness of results

The measure of pathogen fitness, (9), was derived from the reduced model (3), not the full model (1). Therefore, the results from the previous section have been produced under the quasi‐equilibrium assumption. This assumes that the rate of change of viral particles is much faster than the rate of change of susceptible and infected chickens; it assumes that δ and κ are large relative to the other parameter values.

In Figure (4), we explore the impact the quasi‐equilibrium assumption has on the accuracy of the predictions made with (9). We ran a series of simulations with the original model (1), while violating the quasi‐equilibrium assumption. To determine the most successful strain, we found the strain that could withstand invasion by any other strain. For example, we would fix the cohort duration, say *T* = 10 days (as in Figure 5a), and let a wild‐type strain go to an endemic state. Once the wild‐type strain reached a stable periodic orbit, a rare mutant strain is introduced (see Figure 2 for example). If the rare mutant overtook and replaced the wild‐type strain, it would become the new wild‐type strain. If the mutant failed to replace the wild‐type strain, then the wild‐type strain successfully avoided invasion. We continue to do this for 1,000 random mutant strains of MD, each one either successfully invading and becoming the new wild‐type strain, or not. After investigating the 1,000 strains, we are left with the most successful strain of the 1,000 investigated, and this is a good approximation for the ESS for *T* = 10. Once we find the ESS for *T* = 10, we fix a new value of *T* and repeat the procedure.

Eventually, as can be seen in Figure 5, we arrive at a trend similar to that found analytically with (9); as *T* increases, v* decreases. We repeat this procedure for a range of different flock sizes, *N*, as well as for a range of cleaning efforts, γ. Again, the simulation results are qualitatively consistent with those found analytically; as *N* or γ increases, v* decreases.

## 4 Discussion

Over the past 60 years, MD has experienced continual evolution towards greater virulence. The only defence against the very lethal disease is vaccination. The most recent generation of vaccines are highly effective, but there is growing concern that they will lose efficacy and the industry will experience extensive losses as it has historically (Davison & Nair, 2005; Nair, 2005).

In this paper, we derive an impulsive differential equation model that models the spread of two strains of MDV on a single broiler farm, over an indefinite number of cohorts. We found that when the cohort duration, *T*, or the density of chickens, *N*, are increased, evolution favours less virulent strains of the virus. We also found that as the number of viral particles that survive the intercohort period increases, modelled with γ, evolution favours less virulent strains of the virus.

An increase in carry‐over of viral particles from one cohort to the next could result from a decrease in cleaning of the barn or a decrease in time between cohorts. The longer a barn remains empty, the longer is the period of time that the MDV has to decay. According to our model, substandard cleaning of the barn between cohorts and hurried restocking will favour less virulent strains of the virus. As the number of viral particles that remain in the barn between cohorts increases, the effective death rate due to cleaning and loss during the intercohort period decreases, and thus, the relative cost the virus pays for host mortality increases.

Short intercohort times have a number of benefits, one of which is economic; farms profit only when they stock chickens. In addition to economic benefits, short intercohort times, and less intense cleaning of the barn, may benefit chicken health. Introducing newly hatched chicks into a dirty broiler farm has the benefit of exposing them to healthy bacteria left behind in the chickens faeces from the previous cohort. This could help develop a rich microflora of the intestinal tract that promotes health and acts as a barrier to harmful bacteria like salmonella (Nurmi & Rantala, 1973), a bacteria which infects both chickens and humans. There is however a risk of also infecting the very susceptible chicks with harmful pathogens left behind in the dirty barn, so care would have to be taken if changes in management were to happen.

The cohort durations for broilers have only gotten shorter over the years. Advances in nutrition and selection have almost halved the average broiler cohort duration from what it was 60 years ago (Anthony, 1998). We found that increasing the cohort duration can select for lower virulence strains of MDV, a result also found by Atkins et al. (2012), and supported by the historical trend towards greater virulence as cohort durations have shortened (Atkins et al., 2012). As the cohort duration increases, the lifespan of the chicken increases, and so too does the lifespan of the parasites exploiting the host. Therefore, as cohort duration increases, the cost of host death increases, and selection will favour less virulent strains of MD.

Today broiler farms can hold tens of thousands of chickens at a single time (Sheppard, 2004). Intuition might suggest that overcrowded barns would select for more virulent strains of MD; the cost of host death would be small due to the abundance of hosts. However, we found that as the density of chickens in a barn increases, evolution will select for less virulent strains of MD. This is due to the increase in lifespan of the pathogen that results from an increase in *N*. As the density increases (i.e., *N* increases), more viral particles make it through the intercohort period, resulting in a longer lifespan for the pathogen on average. As this “effective” lifespan of the parasite increases, host mortality becomes more costly, and hence, the evolutionary stable virulence level decreases. This increased exploitation time selects for less virulent viral strains that are able to exploit the host for a longer period of time. While increasing the density helps fight the evolution towards greater virulence, Atkins et al. (2013) found that increasing the stocking density increases the probability of a MDV outbreak, while Rozins and Day (2016) found that an increase in stocking density made MD eradication from a barn more difficult to achieve.

If eliminating disease from a barn is the objective, then long cohort durations, densely populated barns and dirty barns are the exact opposite of what you want. Although MD spread can be prevented with extreme cleanliness practices and extensive air filtration (Grunder, Gavora, Spencer, & Turnbull, 1975; Rozins & Day, 2016), it may not be economically feasible at a larger scale. If disease elimination is not possible through intensive cleaning of the barn between cohorts, it may select for more virulent strains of the virus as we have shown here. Similarly, increasing either the population density or cohort duration will make disease elimination on a farm more difficult (Rozins & Day, 2016).

Assumptions were made in the analysis of model (1), one being the quasi‐equilibrium assumption. The impact this assumption has on the mutant‐free, stable periodic orbit (see Figure 1) is discussed in Rozins and Day (2016). The quasi‐equilibrium assumption was also necessary in the derivation of (9), which is a measure of pathogen fitness, and from which our analytical results are based. To explore whether the quasi‐equilibrium assumption affects the results derived from (9), we ran a series of simulations while allowing δ and κ to be small (i.e., a violation of the quasi‐equilibrium assumption). We found that for both small and large δ and κ, the results from Section 2 hold, an increase in cohort duration, *T*, and/or flock size, *N*, or a decrease in intercohort cleaning, (increase in γ), all select for less virulent strains of MD (see Figure 5).

Another assumption made in our analysis has to do with the shape of the transmission–virulence trade‐off curve. The shape of the transmission–virulence trade‐off is not known. Although there has been an upsurge in support that transmission increases with virulence (Alizon et al., 2009), whether the curve is concave up, or concave down, is of debate. Our results rely on the curve being concave down, and given the biological constraints on the parameters, we feel that this assumption is reasonable. Our assumption is that as virulence increases, there is a limit to the number of inclusion bodies the host can support, and thus, transmission also has an upper limit, and thus, the curve is concave down, approaching a horizontal asymptote. Atkins et al. (2012), in a study of MDV evolution, found that fitness was maximized at intermediate virulence levels, also in accord with the standard virulence–transmission trade‐off (Atkins et al., 2012). We should note that alternative assumptions on life‐history traits could have been made. For example, others have assumed a trade‐off between virulence and the rate at which free‐living viral particles decay outside the host (Caraco & Wang, 2008).

One significant difference between the model presented in this paper, and other models exploring virulence evolution, is that we allow for cohorts of limited duration. This explicitly allows us to study the impact cohort duration and intercohort dynamics have on evolution. In fact, often infectious disease models with both continuous dynamics describing the “within‐cohort” period, and discrete dynamics describing an “intercohort” period, assume that the within‐cohort period is of infinite duration (Dwyer, Dushoff, Elkinton, & Levin, 2000; May, 1985). While these other models appear very similar to ours, the resulting dynamics are vastly different (Dwyer et al., 2000; May, 1985). The significant difference is that the MD model presented in this paper has a fixed population size at the start of every cohort. In general, host–pathogen populations in the wild are not constrained in the same way that domesticated livestock–pathogen populations are. Farmers have complete control over the number of livestock occupying their farm at a given time. Unlike domesticated livestock, populations in the wild are at the mercy of available resources, pathogens and predators, and as a result may grow or shrink. Mathematically, this results in our model converging to a stable periodic orbit, while these other similar models exhibit chaotic behaviour.

We have seen the continuous evolution in virulence and an ability to overcome the vaccine‐induced immune response with each newly introduced vaccine. Vaccines are providing only a temporary relief and eventually a viral strain able to bypass the vaccine‐induced immune response will emerge. Slowing evolution, as opposed to investment in new vaccines, has been shown to be more effective at combating the emergence of new resistance pathogens (McClure & Day, 2014). In this paper, we show that the evolution towards higher virulence can be slowed with longer cohort durations, larger flock sizes and less intensive cleaning of the barn.
