# Supplementary material for: The industrialization of farming may be driving virulence evolution
Source: Evol Appl. 2016 Nov 21;10(2):189–98. doi: 10.1111/eva.12442 (PMC5253429; doi:10.1111/eva.12442)
Supplement: Supplementary file 1 [file EVA-10-189-s001.pdf]

[28] Witter, R.L. (1997), "Mareks disease: a worldwide problem." *Avian Diseases*, 41(1), 149–163.

[29] Witter, R.L. (2001), "Marek's disease vaccines - past, present and future. in: Current progress on marek's disease research, proceedings of the 6th international symposium on marek's disease." *American Association of Avian Pathologist, Pennsylvania*, 1–9.

[30] Witter, R.L., G.H. Burgoyne, and B.R. Burmester (1968), "Survival of marek's disease agent in litter and droppings." *Avian diseases*, 522–530.

[31] Witter, R.L., J.I. Moulthrop Jr, G.H. Burgoyne, and H.C. Connell (1970), "Studies on the epidemiology of marek's disease herpesvirus in broiler flocks." *Avian diseases*, 255–267.

# 5 Appendix: ESS

Recall that the evolutionary stable virulence level will maximize:

$$\frac{\beta(v)}{v - \frac{\ln(k\beta(v))}{T}}$$

By taking the derivative of the above with respect to $v$, we can find an expression for $\frac{d\beta(v^*)}{dv}$, the slope of the transmission rate as a function of the evolutionary stable virulence level $v^*$.

$$\frac{d\beta(v^*)}{dv} = \frac{\beta(v^*)}{v^* - \frac{1}{T}\ln(k\beta(v^*)) + \frac{1}{T}}$$

## 5.1 $dv^*/dT$

First we rewrite (10) as a function of $v$ and $T$ and we let this function be denoted $R(v, T)$.

$$R(v,T) = \frac{\beta(v)}{v - \frac{\ln(k\beta(v))}{T}}.$$



For Peer Review

Let $R_v(v,T) = F(v^*,T)$

$$F(v^*,T) = \frac{\frac{d\beta(v^*)}{dv}(v^* - \frac{1}{T}\ln(k\beta(v^*))) - \beta(v^*)(1 - \frac{1}{T\beta(v^*)}\frac{d\beta(v^*)}{dv})}{(v^* - \frac{1}{T}\ln(k\beta(v^*)))^2}$$

If we differentiate $F(v^*,T)$ with respect to $T$ and then substitute in $\frac{d\beta(v^*)}{dv}$ we get:

$$F_T(v^*,T) = -\frac{\beta(v^*)T(\ln(k\beta(v^*)) - 1)}{(-v^*T + \ln(k\beta(v^*)))^2(-v^*T + \ln(k\beta(v^*)) - 1)} < 0$$

If we differentiate $F(v^*,T)$ with respect to $v$ and then substitute in $\frac{d\beta(v^*)}{dv}$ we get:

$$F_{v^*}(v^*,T) = -T\frac{A+B+C}{D} < 0$$

provided $\frac{d^2\beta}{dv^{*2}} < 0$.

where:

$$A = -\frac{d^2\beta(v^*)}{dv^{*2}}T^3v^{*3} + 3T^2v^{*2}\frac{d^2\beta(v^*)}{dv^{*2}}\ln(k\beta(v^*)) - 3T^2v^{*2}\frac{d^2\beta(v^*)}{dv^*} + \frac{d^2\beta(v^*)}{dv^*}(\ln(k\beta(v^*)))^3 > 0$$

$$B = 6Tv^*\frac{d^2\beta(v^*)}{dv^{*2}}(\ln(k\beta(v^*))) + \beta(v^*)T^2 > 0$$

$$C = -3\frac{d^2\beta(v^*)}{dv^{*2}}(\ln(k\beta(v^*)))^2 - 3\frac{d^2\beta(v^*)}{dv^{*2}}v^*T + 3\frac{d^2\beta(v^*)}{dv^{*2}}\ln(k\beta(v^*)) - \frac{d^2\beta(v^*)}{dv^{*2}} > 0$$

and

$$D = (-v^*T + \ln(k\beta(v^*))))^2(-v^*T + ln(k\beta(v^*)) - 1)^2 > 0$$

Finally if we implicitly differentiate $F(v^*,T) = 0$ with respect to $T$ and treat optimal virulence, $v^*$, as a function of cohort duration, $T$, we get:

$$F_{v^*}(v^*,T)\frac{dv^*}{dT} + F_T(v^*,T) = 0$$

From which we have,

For Peer Review

$$\frac{dv^*}{dT} = -\frac{F_T(v^*,T)}{F_{v^*}(v^*,T)} < 0$$

## 5.2 $dv^*/dk$

Next we rewrite (10) as a function of $v$ and $k$ and we let this function be denoted $S(v,k)$. We let $G(v^*,k) = S_v(v^*,k)$ and treat the optimal virulence level, $v^*$, as a function of $k$.

$$G(v^*,k) = S_v(v^*,k) = R_v(v^*,T) = \frac{\frac{d\beta(v^*)}{dv}(v^* - \frac{1}{T}\ln(k\beta(v^*))) - \beta(v^*)(1 - \frac{1}{T\beta(v^*)}\frac{d\beta(v^*)}{dv})}{(v^* - \frac{1}{T}\ln(k\beta(v^*)))^2}$$

and by differentiating $G(v^*,k)$ with respect to $k$ and substituting in $\frac{d\beta(v^*)}{dv}$ we get:

$$G_k(v^*,k) = \frac{\beta(v^*)T^2(k+1)}{k(-v^*kT + k\ln(k\beta(v^*)) - 1)(-v^*T + \ln(k\beta(v^*)))^2} < 0$$

Finally if we implicitly differentiate $G(v^*,T) = 0$ with respect to $k$ and treat optimal virulence, $v^*$, as a function of cohort duration, $k$, we get:

$$G_{v^*}(v^*,T)\frac{dv^*}{dk} + G_k(v^*,T) = 0$$

From which we have,

$$\frac{dv^*}{dk} = -\frac{G_k(v^*,k)}{G_{v^*}(v^*,k)} < 0$$

For Peer Review
